# ESMI: a macrophyte index for assessing the ecological status of lakes

**DOI:** 10.1007/s10661-014-3799-1

**Published:** 2014-05-18

**Authors:** Hanna Ciecierska, Agnieszka Kolada

**Affiliations:** 1 Department of Botany and Nature Protection, University of Warmia and Mazury, Plac Łódzki 1, Kortowo, 10-727 Olsztyn, Poland; 2 Department of Freshwater Assessment Methods and Monitoring, Institute of Environmental Protection-National Research Institute, Kolektorska 4, 01-692 Warsaw, Poland

**Keywords:** ESMI, Macrophytes, Assessment method, Field survey, Water Framework Directive

## Abstract

The paper introduces the Ecological State Macrophyte Index (ESMI), a method compliant with the Water Framework Directive (2000/60/EC) for assessing the ecological status of lakes based on macrophytes. A description of the elaboration of macrophyte metrics, relevant reference conditions and a classification system for two types of high-alkalinity lowland lakes (stratified and polymictic), and a customised field survey procedure based on belt transects are presented. The ESMI evaluates two aspects of macrophyte community: taxonomic composition (index of evenness *J*) and abundance (colonisation index *Z*), which are combined into one multimetric. ESMI values range from 0 to 1, where 1 denotes pristine conditions and 0, highly degraded habitats. The high/good class boundary (*H*/*G*) was set at the first quartile of ESMI values of reference lakes. For the other classes, boundaries were set by dividing the range of ESMI values between the *H*/*G* boundary and the minimum value recorded in the dataset in a logarithmic scale into four. The ESMI correlated best with water transparency (Pearson’s *R* = 0.62 in stratified lakes and 0.79 in polymictic ones), whereas the correlations with phosphorus and nitrogen concentrations were somewhat weaker (*R* = −0.48 to −0.57). Based on the results of international intercalibration, the original class boundaries were modified (merged for stratified and polymictic lakes, the good/moderate boundary tightened to approximately 20 %), to make ESMI-based assessment results comparable with the outcomes of other European methods.

**Electronic supplementary material:**

The online version of this article (doi:10.1007/s10661-014-3799-1) contains supplementary material, which is available to authorized users.

## Introduction

The role of the phytolittoral zone in lake functioning is well known and has been emphasised by many authors. Macrophytes offer refuge and food for small animals, drive nutrient dynamics in an ecosystem, prevent sediment resuspension and release oxygen during photosynthesis (synthetic overview, e.g. in Jeppesen et al. 1998; Scheffer 1998; Pokorný and Kvĕt 2004). The health of aquatic vegetation and biological processes in the phytolittoral zone are vital for the functioning of lake ecosystems. Aquatic plants integrate temporal, spatial, chemical, physical and biological characteristics of an ecosystem, and their distribution and abundance are influenced by variations in environmental conditions (Lacoul and Freedman 2006). Macrophytes respond to changes in the environment and are reliable indicators of ecosystem health (Palmer et al. 1992; Robach et al. 1996; Dawson et al. 1999; Melzer 1999; Smolders et al. 2001; Haury et al. 2006; Schneider 2007). Nonetheless, until the beginning of the twenty-first century, bioassessment protocols have been limited in many European countries and aquatic vegetation has not been a major issue in most monitoring programmes (Knoben et al. 1995).

The effective management of water resources requires a comprehensive understanding of ecosystem functions and interactions. The ecological approach to water management in Europe was implemented in 2000 in the form of the European Commission Water Framework Directive (WFD, EC 2000). The overriding goal of the WFD is to bring aquatic habitats to “good ecological status”, defined as a slight deviation from undisturbed (reference) conditions, with no or minor human impact. The ecological status is evaluated based on various indicators of biological quality, including phytoplankton, macrophytes, phytobenthos, benthic invertebrates and fish fauna. Assessments are based on the Ecological Quality Ratio (EQR), which indicates the relationship between the values of biological parameters observed in a water body and in reference conditions applicable to that water body. The reference conditions should be type-specific, and they should reflect the diversity of biological communities, which is determined by given abiotic conditions. As a part of the typological approach, the development of similar biological communities is assessed based on similar physical and chemical descriptors, and different benchmarks are used to evaluate, e.g. highly alkaline lowland lakes in a temperate climate and oligotrophic lakes in the Alpine region.

Most assessment systems existing in the year 2000 in the EU were not compliant with the WFD, as they were not reference-based or specific to water types (Hering et al. 2010). The inception of the WFD has stimulated the intensive development and improvement of an array of bioassessment methods across the EU during the last decade (Birk et al. 2012; Lyche Solheim et al. 2013). For biological monitoring, it is crucial to develop a method that supports comprehensive, quick and cost-effective surveys and generates highly accurate data for reliable and unambiguous assessments of the ecological status of water bodies. Furthermore, the method should meet the specific criteria mentioned above to ensure its compliance with WFD requirements.

Prior to the introduction of the WFD, lake quality evaluation and management in Poland have focused mainly on pollution control and macrophytes have not been included in monitoring programmes. In this paper, we present the Polish WFD-compliant macrophyte-based method for lake assessment, the Ecological State Macrophyte Index (ESMI), which was developed in 2006 within a dedicated project commissioned by the Ministry of Environment. Our study includes: (1) adaptation of the original Polish macrophyte method (Rejewski 1981) to WFD requirements; (2) establishing the reference conditions and classification system for two types of temperate lowland hard-water lakes, stratified and polymictic; (3) modification of the original sampling procedure used in the method by Rejewski (1981) and its adaptation to routine lake monitoring; (4) validation of the ESMI method after the first 6 years of application in routine lake monitoring in Poland; (5) harmonisation of the ESMI class boundaries according to the recommendations derived from the pan-European intercalibration process (EC 2005).

## Materials and methods

### Historical background—methodological concept

As the core concept underlying the WFD-compliant macrophyte method in Poland, the Polish method developed in the late 1970s by Rejewski (1981), here referred to as the macrophytoindication method (MPhI), was adopted. This method involves a phytosociological approach (Braun-Blanquet 1964), where the characterisation of the vegetation is based on phytosociological units—plant communities. The term ‘community’ denotes areas of homogenous and uniform vegetation areas (phytocenoses sensu Westhoff and van der Maarel 1973, after Jensén 1977), named after the predominant species. As a part of the MPhI, the synanthropisation index *I*_s_ was elaborated, which evaluates the synanthropisation of lake vegetation, defined as the degree of simplification of the taxonomic composition and spatial structure of biotic communities resulting from anthropogenic pressure. The index is a ratio of redundancy index *R* (Eqs. 1, 2, 3) and colonisation index *Z* (Eq. 4), where:

1 $$ R=1-\frac{H}{H_{\max }} $$

2 $$ H=-\sum \frac{n_i}{N}\times \ln \frac{n_i}{N} $$

3 $$ {H}_{\max }= \ln S $$

where *n*_i_ is the proportion of the lake area inhabited by each plant community in the total area of the phytolittoral, *N* is the total area vegetated (100 %) and *S* is the total number of plant communities; and:

4 $$ Z=\frac{N}{P_{isob2.5}} $$

where *N* is the total phytolittoral area and *P*_isob2.5_ is the potential phytolittoral area bounded by the 2.5 m isobath (lake area with a depth of less than 2.5 m). The values of *I*_S_ can vary within wide limits; the higher the *I*_S_, the more altered and anthropogenic is the structure of the lake vegetation. *I*_S_ allows the classification of a lake to the one of five classes of synanthropisation of aquatic vegetation, where an *I*_S_ value in a range from 0.0 to 0.25 denotes near natural conditions and an *I*_S_ > 1.0 indicates the most degraded ecosystems.

Over the years, the method has been further developed and modified by Ciecierska (2004a, b, c, 2006, 2008), and within this study, it was fully adapted to WFD requirements as the Ecological State Macrophyte Index (ESMI).

### Data collection

The adaptation of the MPhI method was aimed at designing a method to be used in national lake monitoring, which in Poland is focused primarily on water bodies of an area greater than 0.5 km^2^. The vast majority of Polish lakes that fall into the above category are small- to medium-sized (0.5–10.0 km^2^) lowland water bodies (<200 m a.s.l.) with a high-alkalinity and non-coloured waters (>1.0 meq/L, <30 mg Pt/L), and they account for 97 % of Polish lakes larger than 0.5 km^2^ (Kolada et al. 2005). Soft-water lakes and coastal lakes are very specific and rare in Poland (26 lakes and 9 lakes larger than 0.5 km^2^, respectively, Kolada et al. 2005). Therefore, in our study, only hard-water lowland lakes were analysed. As macrophyte monitoring was not conducted in Poland prior to the WFD, the only source of information concerning aquatic vegetation in our study was research data. The research data available from lakes larger than 0.5 km^2^ were very limited, thus, we expanded our dataset to include smaller lakes with an area of >0.2 km^2^.

**Fig. 1:**
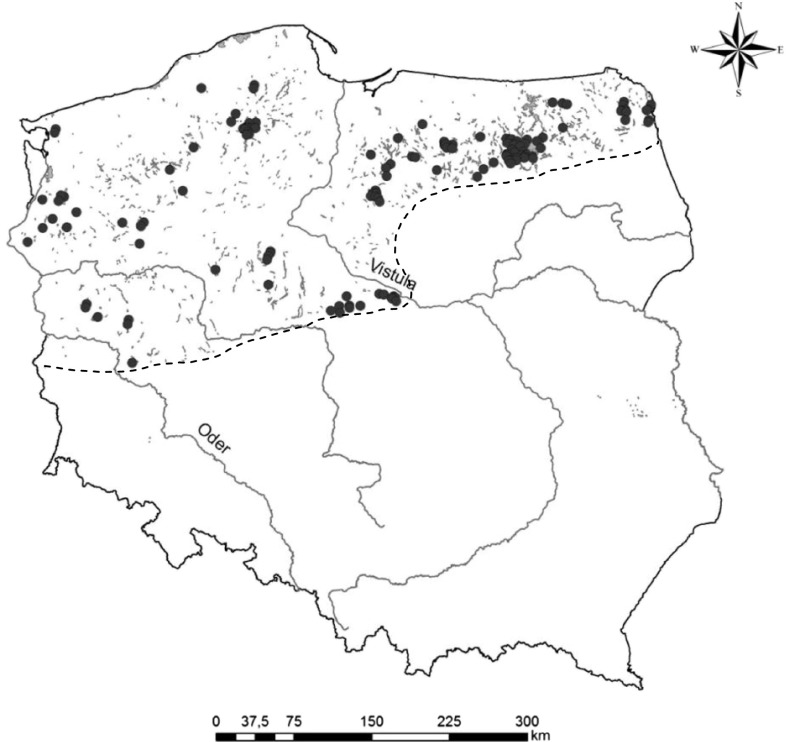
Geographical distribution of lakes in Polish lake districts. The lakes analysed in the study are marked with *circles* (*n* = 138); *dashed line* limit of the last glaciation (the area comprising the majority of Polish natural lakes larger than 1 ha)

Data on aquatic and rush vegetation from 138 lakes surveyed in the years 1970–2006 (165 lake-years constituting independent data; 5 to 25 years between subsequent surveys for lakes investigated more than once except for two lakes, Majcz Wielki and Kołowin, which were investigated more frequently; Appendix 1 in Online Resource 1) were used to adapt the MPhI method to WFD requirements. Biological data from 127 lake-years have been collected by the authors during research projects since the 1980s; data from 38 lake-years were derived from a review of the literature. A list of lakes and references are provided in Appendix 1. For 89 lake-years (54 %), the monitoring data on the main eutrophication indicators, total phosphorus (TP), total nitrogen (TN), chlorophyll *a* concentrations (Chl*a*) and Secchi depth (SD), were available. These were collected using a pre-WFD sampling procedure, i.e., twice a year, in spring and summer, in the same year (32 lake-years) or not earlier/later than three (45), occasionally five (12) years from a macrophyte survey (Appendix 1). The analysed lakes are evenly distributed in Polish lake districts (Fig. 1) and they are all lowland, hard-water ecosystems located within the limit of the last glaciations on postglacial deposits, and represent different morphometric and hydrographic conditions and considerable variations in water quality (Table 1).

**Table 1:** The main hydromorphological characteristics and spring and summer means of the main eutrophication parameters in 138 lakes which were used to develop the macrophyte-based method ESMI

Lake features (unit)	Mean	St.dev.	Range
Area (km^2^)	1.78	3.56	0.20–35.3
Mean depth (m)	5.8	3.9	0.6–23.3
Maximum depth (m)	16.8	13.6	1.7–68.0
Volume (10^3^ m^3^)	15,503.7	61,380.2	169.2–681,672.4
Colour (mgPt/L)	15	7	3–30
Alkalinity (meq/L)	2.6	0.5	1.9–4.0
Conductivity (μS/cm)	463	535	200–3,490
pH	8.4	0.5	6.8–9.5
TP (mgP/L)	0.118	0.214	0.020–1.258
TN (mgN/L)	1.45	1.01	0.25–5.61
Chl*a* (μg/L)	26.8	29.7	0.9–119.2
SD (m)	2.10	1.35	0.20–5.40

*st.dev.* standard deviation, *TP* total phosphorus, *TN* total nitrogen, *Chla* chlorophyll *a* concentrations, *SD* Secchi depth

Biological data used in the study were collected using a unified sampling procedure, which relies on a phytolittoral inventory. A detailed carpet mapping of the entire phytolittoral zone was carried out by multiple dense sampling with a rake or grapnel and bathyscope observations. Hard-copy bathymetric maps were used in the field to determine the spatial ranges and depth distributions of submerged, floating-leaved and emergent plant communities inhabiting the littoral zone. Bathymetric data were used to calculate the area occupied by each plant community. The phytosociological approach was applied (Braun-Blanquet 1964) to identify and classify aquatic and rush vegetation.

### Elaboration of the WFD-compliant method

The adaptation of the MPhI method was performed towards ensuring that the new method: (1) includes taxonomic composition and abundance of macrophytes; (2) refers to type-specific reference conditions; (3) produces a result that falls within a range from 0 to 1, where 1 describes nearly pristine conditions and 0, the most disturbed conditions; (4) allows the classification of a lake into one of five classes of ecological status. The *I*_S_ formula, proposed by Rejewski (1981), was modified accordingly, to ensure the index met all the above criteria. Moreover, the index should respond clearly and directionally to pressure. In our study, eutrophication was addressed as the main pressure affecting Polish lakes. The performance of ESMI was tested against the seasonal mean of the water quality indicators (TP, TN and SD) using Pearson’s correlation analysis. Due to the high inter-correlation between SD and Chl*a* (Pearson’s *R* = 0.85, *p* < 0.001), the response of ESMI to the latter was not analysed. Water quality data were log-transformed prior to the analyses. Transformation did not improve the distribution of macrophyte variables, thus, they remained untransformed.

To define the reference conditions for Polish lakes, a spatially based approach (“the best of existing”), in which data from undisturbed or only minimally disturbed lakes are analysed (EC 2003), was adopted. A set of pressure-screening criteria was used to select potential reference lakes (an absence of urban areas, low human population density, a high proportion of forests and wetlands in catchments, an absence of human settlements in the direct vicinity of the shoreline, an absence or very low recreational use), together with water quality indicators (high water quality according to the scoring scheme Lake Quality Evaluation System used in pre-WFD lake monitoring; Kudelska et al. 1997) and biological parameters (mean seasonal chlorophyll *a* concentrations below 10 μg/L and no evidence of aquatic vegetation deterioration). The frequency of plant communities in reference and non-reference lakes was analysed, and the distributions of macrophyte variables and water quality indicators were compared between reference and non-reference lakes in stratified and polymictic lakes, separately. Due to considerable differences in the sample size between the compared groups, the Mann–Whitney *U* test was applied.

The boundary values of ecological status classes were determined based on the distribution of ESMI values in the studied lakes, separately for stratified and polymictic lakes. The high/good class boundary (*H*/*G*) was set at the first quartile of ESMI values for reference lakes. In the remaining classes, boundaries were set by dividing the range of ESMI values between the *H*/*G* boundary and the minimum value recorded in the dataset in logarithmic scale into four. The distribution of eutrophication indicator values and means across ecological status classes was tested using ANOVA.

The original field survey procedure, which involves a complete phytolittoral inventory, is detailed and relatively precise, but is also a highly strenuous and time-consuming technique. Hence, to reduce the sampling effort, a less laborious procedure with a higher benefit–cost ratio was designed and recommended for routine lake monitoring. The accuracy of the proposed belt transect method and the original mapping method was tested by comparing the distribution of macrophyte metric values produced by both sampling techniques for 13 lakes surveyed in 2006 using *t* tests and regression analysis. All statistical procedures were performed using the STATISTICA 7.1 software (StatSoft, Inc 2005).

### Validation of the ESMI method

In the years 2009–2011, the ESMI method was subjected to international comparison known as “intercalibration exercise” (EC 2005). The requirement of intercalibration of EU methods was introduced by Annex V of the WFD, to ensure that the normative definitions for the high and good quality of surface water are interpreted equally across Europe, regardless of differences in ecological quality assessment systems between the Member States (i.e., the good ecological status represents the same level of ecological quality everywhere in Europe). The detailed procedure and results of intercalibration of lake macrophyte-based methods of Central-Baltic European region are described in Portielje et al. (2014). Here, we briefly present and discuss the consequences of the results of a pan-European intercalibration process for macrophyte-based lake classification in Poland.

In 2013, the effectiveness of the ESMI index was validated using an independent dataset that has been collected since 2007 during routine lake monitoring. Biological and environmental data from 427 lakes surveyed in 2007–2012 were used to evaluate the correlations between ESMI (macrophytes surveyed once in the growing season) and seasonal mean values of water quality indicators (TP, TN and SD; water samples collected three times during the growing season; data log-transformed).

## Results: presentation of the ESMI method

### Metrics on taxonomic composition and abundance

In line with WFD requirements, the ESMI method evaluates two main aspects of macrophyte community: taxonomic composition and abundance. The ratio of the biocenotic diversity index H (Shannon and Weaver 1949) and the maximum biocenotic diversity index *H*_max_ included in the redundancy index *R* (Eq. 1) was employed as the taxonomic composition component. This ratio is known as Pielou’s index of evenness *J* (Pielou 1975). The colonisation index *Z* (Eq. 4) was accepted as the basic measure of macrophyte abundance. These two components were combined into one multimetric—the ESMI (Eq. 5):

5 $$ \mathrm{ESMI}=1- \exp \left[-J\times Z\times \exp \left(\frac{N}{P}\right)\right] $$

When the calculated value is subtracted from 1 and an exponential function is introduced, the function approaches the horizontal asymptote of 1, and ESMI values are determined in the range of 0 to 1, where 1 is indicative of pristine ecosystems (reference status) and 0 denotes highly degraded habitats. The typological factor exp(*N*/*P*) (where exp is natural exponential function with Euler’s number *e* as a base, *N* is the total vegetated area and *P* is the lake area) has been included to prevent downgrading of lakes with a naturally very low colonisation depth, which is determined by lake morphology rather than water quality, i.e., very shallow lakes with a mean depth <2.5 m, where the colonisation index *Z* <1.0.

In both stratified and polymictic lakes in the research database, ESMI correlated significantly with all water quality parameters tested, best and positively with SD (*R* = 0.62 in stratified and 0.79 in polimictic lakes) and slightly weaker and negatively with TP and TN. In polymictic lakes, ESMI responded stronger to TN than to TP, whereas in stratified lakes, the relationships between ESMI and both nutrients were similar (Table 2).

**Table 2:** The Pearson’s correlations *R* between ESMI and the seasonal means of the main water quality parameters in Polish lowland lakes used to develop (historical and research data from the period 1970–2006; *n* = 89) and to verify (monitoring data from the period 2007–2012; *n* = 427) the ESMI method

Lake type	Historical and research data (1970–2006)						Monitoring data (2007–2012)					
	LogTP		LogTN		LogSD		LogTP		LogTN		LogSD	
	*R*	*p*	*R*	*p*	*R*	*p*	*R*	*p*	*R*	*p*	*R*	*p*
Stratified (*n* = 62^a^/241^b^)	−0.50	<0.001	−0.48	<0.001	0.62	<0.001	−0.48	<0.001	−0.49	<0.001	0.62	<0.001
Polymictic (*n* = 27^a^/186^b^)	−0.52	0.008	−0.57	0.002	0.79	<0.001	−0.32	<0.001	−0.50	<0.001	0.70	<0.001

*TP*, *TN*, *SD* refer to the key in Table 1

^a^Number of lake-years in historical and research dataset

^b^Number of lake-years in the monitoring dataset

### Reference conditions

Due to its mathematical structure (exponential function), the maximum value of ESMI approaches the horizontal asymptote equal to 1 which constitutes the theoretical reference value. However, to derive the (*H*/*G*) boundary value (set at the first quartile of the ESMI range for reference lakes), reference conditions have to be identified.

Based on pressure and biological criteria, 26 reference lakes were selected, including 18 stratified and 8 polymictic lakes. In lakes of both types, in the reference state, the most frequently occurring hydrophytes (found in >60 % of lake-years) were *Chara tomentosa*, *Chara rudis*, *Nitellopsis obtusa*, *Potamogeton lucens*, *Potamogeton perfoliatus*, *Nuphar lutea* and *Stratiotes aloides*, and in stratified lakes, also *Chara fragilis*, *Myriophyllum spicatum* and *Ranunculus circinatus* (Appendix 2 in Online Resource 2). The most abundant hydrophytes, covering >10 % of the total phytolittoral area on average, were *C. tomentosa*, *C. rudis* and *N. obtusa*, and in stratified lakes, also *Chara delicatula* and *C. fragilis*. In general, non-disturbed habitats were characterised by a predominance of stonewort communities which, when compared with vegetation patterns in non-reference lakes, emerge as a distinctive feature of reference conditions in Polish lowland lakes (Appendix 2, Table 3).

**Table 3:** The medians and range (given in brackets) of the main variables of aquatic vegetation, macrophyte metrics and water quality indicators in reference (REF) and non-reference (NON-REF) lowland (<200 m a.s.l.) hard-water (>1 meq/L) lakes, stratified and polymictic

Variable group	Variable	Stratified lakes				Polymictic lakes			
		REF *n* = 18^a^/15^b^	NON-REF *n* = 78^a^/47^b^	*U* test		REF *n* = 8^a^/5^b^	NON-REF *n* = 61^a^/22^b^	*U* test	
				*U*	*p*			*U*	*p*
Abundance and structure	*S*	24 (14–31)	18 (7–32)	350.5	0.001	24 (15–27)	14 (4–29)	65.5	<0.001
	*C*_max_	5.0 (3.0–7.5)	3.0 (1.0–6.0)	144.0	<0.001	3.8 (2.5–5.0)	2.0 (1.0–6.0)	59.5	0.002
	%*N*	38.8 (14.3–57.0)	18.8 (1.5–73.9)	250.0	<0.001	69.8 (31.5–99.7)	24.5 (0.5–99.7)	95.0	0.005
	%Char	53.0 (25.9–83.8)	0 (0–76.6)	57.0	<0.001	36.1 (17.9–61.4)	0 (0–84.6)	62.0	<0.001
	%Pota	13.9 (0.1–55.5)	33.2 (0–95.1)	305.5	0.008	13.9 (0.4–29.5)	22.3 (0–97.8)	189.5	0.336
	%Nymph	1.3 (0–8.7)	2.2 (0–54.1)	394.0	0.113	7.8 (0.8–16.6)	3.1 (0–50.6)	180.5	0.283
	%Emerg	30.8 (4.3–53.6)	44.6 (2.6–99.9)	311.0	0.010	41.7 (23.4–65.1)	53.6 (1.1–99.6)	172.0	0.216
Macrophyte metrics	*J*	0.59 (0.38–0.79)	0.56 (0.11–0.88)	574.5	0.328	0.59 (0.53–0.71)	0.53 (0.17–0.86)	168.0	0.171
	*Z*	1.74 (1.1–2.22)	0.94 (0.058–1.72)	142.0	<0.001	1.34 (0.95–1.69)	0.65 (0.04–2.22)	75.0	0.002
	ESMI	0.766 (0.447–0.931)	0.436 (0.031–0.850)	173.0	<0.001	0.772 (0.561–0.939)	0.406 (0.027–0.960)	67.0	0.001
Water quality	TP	0.025 (0.020–0.055)	0.057 (0.024–0.378)	118.0	<0.001	0.033 (0.039–0.060)	0.130 (0.028–0.349)	8.0	0.004
	TN	0.77 (0.25–1.40)	1.26 (0.42–4.82)	95.0	<0.001	0.83 (0.58–0.65)	2.11 (1.04–5.61)	10.0	0.005
	Chl*a*	4.0 (0.9–9.0)	16.7 (2.6–104.8)	62.5	<0.001	9.1 (5.2–10.0)	45.1 (17.0–119.2)	5.0	0.002
	SD	3.7 (2.4–5.4)	1.8 (0.6–5.4)	93.0	<0.001	2.5 (2.0–2.5)	0.8 (0.2–1.9)	3.0	0.001

*U* test the Mann–Whitney test used for comparison of the variable distribution between REF and NON-REF lakes, stratified and polymictic

*S* number of plant communities (syntaxa); *C*_max_ maximum colonisation depth (m); *%N* proportion of the phytolittoral area in the total lake area; *%X* proportion of the phytolittoral area occupied by phytocoenoses of stoneworts (%Char), vascular submerged plants (%Pota); floating-leaved plants (%Nymph), emergent plants (%Emerg); *J* Pielou’s index of evenness; *Z* colonization index; *ESMI* Ecological State Macrophyte Index; *TP*, *TN*, *Chla*, *SD* refer to the key in Table 1

^a^Number of lakes with macrophyte data available

^b^Number of lakes with data on water quality available

In both types of lakes, the distribution of macrophyte metrics and water quality indicators differed significantly between reference and non-reference lakes (Mann–Whitney *p* < 0.05). In general, the number of plant communities (*S*), maximum colonisation depth (*C*_max_), share of vegetated lake area (%*N*), share of phytolittoral area covered by stoneworts, *Z*, ESMI and SD values were higher, whereas the share of helophytes, TP, TN and Chl*a* values were lower in reference lakes than in non-reference lakes (Table 3). The only non-significant differences between reference and non-reference lakes were found in the values of index *J* in both lake types and in the proportion of the lake area occupied by elodeids, nympheids and emergent vegetation in polymictic lakes.

**Fig. 2:**
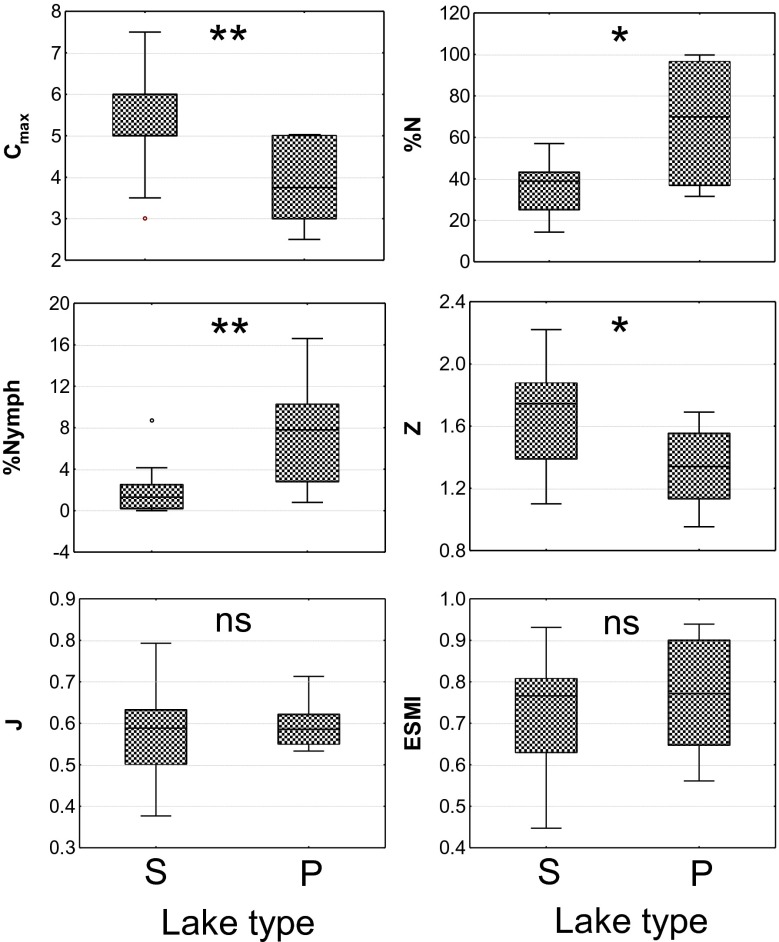
Distribution of selected macrophyte metrics in stratified (*S*, *n* = 18) and polymictic (*P*, *n* = 8) reference lakes; *C*_max_, %*N*, *Z*, *%Nymph* refer to the key in Table 3. *Boxplots* 25–75^th^ percentiles with median, *whiskers* range, *circles* outliers. *Stars* indicate the level of confidence in comparison of metric distribution in reference stratified and polymictic lakes obtained in Mann–Whitney *U* test: ***p < 0.001, **p < 0.01, *p < 0.05, *ns* non-significant

In reference conditions, *C*_max_ (*U* = 18.5, *p* = 0.003) and *Z* (*U* = 31.0, *p* = 0.023) were significantly higher in stratified lakes, whereas %*N* (*U* = 33.0, *p* = 0.030) and the proportion of lake area colonised by floating-leaved vegetation (*U* = 23.5, *p* = 0.009) were significantly higher in polymictic lakes (Fig. 2). All other biological metrics tested, including *J* and ESMI, were not statistically different between the reference stratified and polymictic lakes (*U* = 65.0, *p* = 0.697 for *J* and *U* = 63.0, *p* = 0.617 for ESMI; Fig. 2). Significant variations in ESMI values between reference and non-reference conditions in both stratified (*U* = 173, *p* < 0.001) and polymictic lakes were observed (*U* = 67.0, *p* = 0.001; Table 3).

### Lake classification system

The distribution of ESMI values in stratified and polymictic lakes was analysed to determine boundary values for ecological status classes. As the maximum value of ESMI approaches 1 due to its mathematical structure, a reference value did not have to be determined. The first quartile of ESMI values in reference lakes corresponded to 0.676 in stratified lakes (range between 0.447 and 0.931) and 0.679 in polymictic lakes (0.561 to 0.939; Table 3). The *H/G* class boundary in both lake types was thus set at 0.680. In the remaining classes, boundaries were set by dividing the range of ESMI values between the *H*/*G* boundary and the minimum value recorded in the dataset (0.031 for stratified lakes and 0.027 for polymictic lakes) in a logarithmic scale into four, and the results were rounded to the nearest 0.010 (Table 4).

**Table 4:** Boundary values of the ESMI index for classifying the ecological status of Polish high-alkalinity lowland lakes in the original method developed in 2006 (1) and in the method modified after intercalibration in 2011 (2)

Ecological status	Ranges of ESMI values		
	1		2
	Stratified	Polymictic	All lakes
High	≥0.680	≥0.680	≥0.680
Good	0.340–0.679	0.270–0.679	0.410–0.679
Moderate	0.170–0.339	0.110–0.269	0.205–0.409
Poor	0.090–0.169	0.050–0.109	0.070–0.204
Bad	<0.090	<0.050	<0.070
	or lack of submerged vegetation		

According to the post-hoc assessment performed after the classification system has been elaborated, in a group of non-reference lakes, 21 lakes were assessed as high, 66 as good, 30 as moderate, 12 as poor and 10 as bad (Appendix 1). It revealed that 21 lakes subjected to anthropogenic pressure represented high status based on macrophytes, although those lakes were not considered reference. Moreover, in a group of 26 reference lakes the value of ESMI ranged between 0.447 and 0.939; hence 8 reference lakes with ESMI < 0.680 were not classified as high based on macrophytes. That was a statistical consequence of setting the *H*/*G* boundary at the first quartile of the ESMI value in reference lakes (25 % of the reference lakes are potentially underestimated).

### Customised field survey procedure

The number of plant communities, the proportion of each community in the total vegetation cover and macrophyte spatial colonisation patterns must be identified to define the ecological status of a lake based on ESMI. The phytolittoral mapping used in the original MPhI method is laborious and time-consuming; therefore, to reduce the sampling effort, a belt transect method was proposed for routine monitoring. Observations of aquatic vegetation were performed along 30-m-wide belt transects set perpendicular to the shoreline, where the length of transects covered the entire vegetated area from the upper eulittoral to the outer limit of macrophyte growth. The minimum number of transects was determined by the size and perimeter of a lake based on the method proposed by Jensen (1977) with modified formula (Eq. 6) proposed by Keskitalo and Salonen (1994):

6 $$ \mathrm{NPA}=\left(\frac{T_{\mathrm{mj}}}{2}+\frac{P-{P}_{\mathrm{mj}}}{P_{\mathrm{mj}}}\right)x\frac{L}{\sqrt{\pi \times P}} $$

where NPA is the total number of transects (rounded to the nearest integer), *L* is the length of the shoreline (km), *P* is total lake area (km^2^), *T*_mj_ is the least number of transects required for lakes in a given size class (Table 5) and *P*_mj_ is the lower limit of a given size class (Table 5). Transects should be distributed evenly along the shoreline, but their exact location can be modified, subject to lake morphology and the land-use structure of the surrounding land, to comprehensively illustrate the variability in lake vegetation. All bays, shallow areas, inflows, outflows, changes in bed-slope and land-use types should be taken into account.

**Table 5:** Lake size classification for the determination of the number of macrophyte transects (Jensén 1977; Keskitalo and Salonen 1994, modified)

Size class	*P* (km^2^)	T_min_
I–II	<0.20	2
III	0.20–0.39	2
IV	0.40–0.79	4
V	0.80–1.59	6
VI	1.60–3.19	8
VII	3.20–6.39	10
VIII	6.40–12.79	12
IX	12.80–25.59	14
X	25.60–51.19	16
XI	51.20–102.39	18

*P* lake area, *T*_*min*_ minimum number of transects for each size class

Observations were carried out by wading and boating, using a rake and a bathyscope. Aquatic and rush vegetation was identified and classified using the phytosociological approach (Braun-Blanquet 1964). Each transect served as a synecological relevé, where the relationships among plant communities (not species) was explored. The syntaxonomic systems developed by Brzeg and Wojterska (2001) and Matuszkiewicz (2002) for Polish aquatic and rush vegetation were used. In each transect, maximum colonisation depth and mean vegetation cover were determined, and the communities of submerged, floating-leaved and emergent plants were identified. Total plant cover and relative cover of all plant communities were determined based on the seven-class scale proposed by Braun-Blanquet (1964) (Table 6).

**Table 6:** Cover classes proposed by Braun-Blanquet (1964); mean cover derived from empirical data

Class	Range of cover (%)	Mean cover (%)
5	75–100	86
4	50–75	61
3	25–50	34
2	5–25	15
1	1–5	3
+	0.1–1	0.5
*r*	<0.1	0.1

Total vegetated area (*N*) was derived from *C*_max_ and %cover averaged across all transects and bathymetric data (where the area between subsequent isobaths was given). The proportion of lake area occupied by each plant community (*n*_i_) was recalculated for the entire lake by converting Braun-Blanquet classes into mean percentage cover (as in Table 6) and averaging the results across all the transects. This approach was used to generate input data for calculations of macrophyte metrics.

**Fig. 3:**
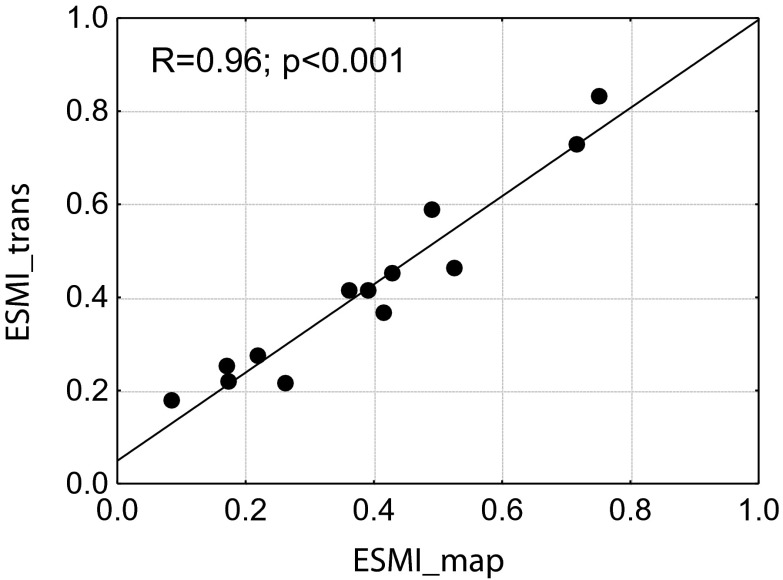
Correlation between ESMI calculated for 13 lakes surveyed in 2006 using the original phytolittoral mapping method (ESMI_map) and the belt transect method (ESMI_trans)

The distribution of all analysed macrophyte parameters (*S*, *C*_max_, %*N*, *J*, *Z*, ESMI) yielded by the two sampling techniques for 13 lakes were not statistically different (*df* = 24, all *p* > 0.20 in *t* test). The Pearson’s correlations between data collected using both techniques ranged between *R* = 0.78 for *J* to *R* = 1.0 for *C*_max_, %N and *Z* (all *p* < 0.001) and was *R* = 0.96 for ESMI (Fig. 3).

### Verification and intercalibration of ESMI

**Fig. 4:**
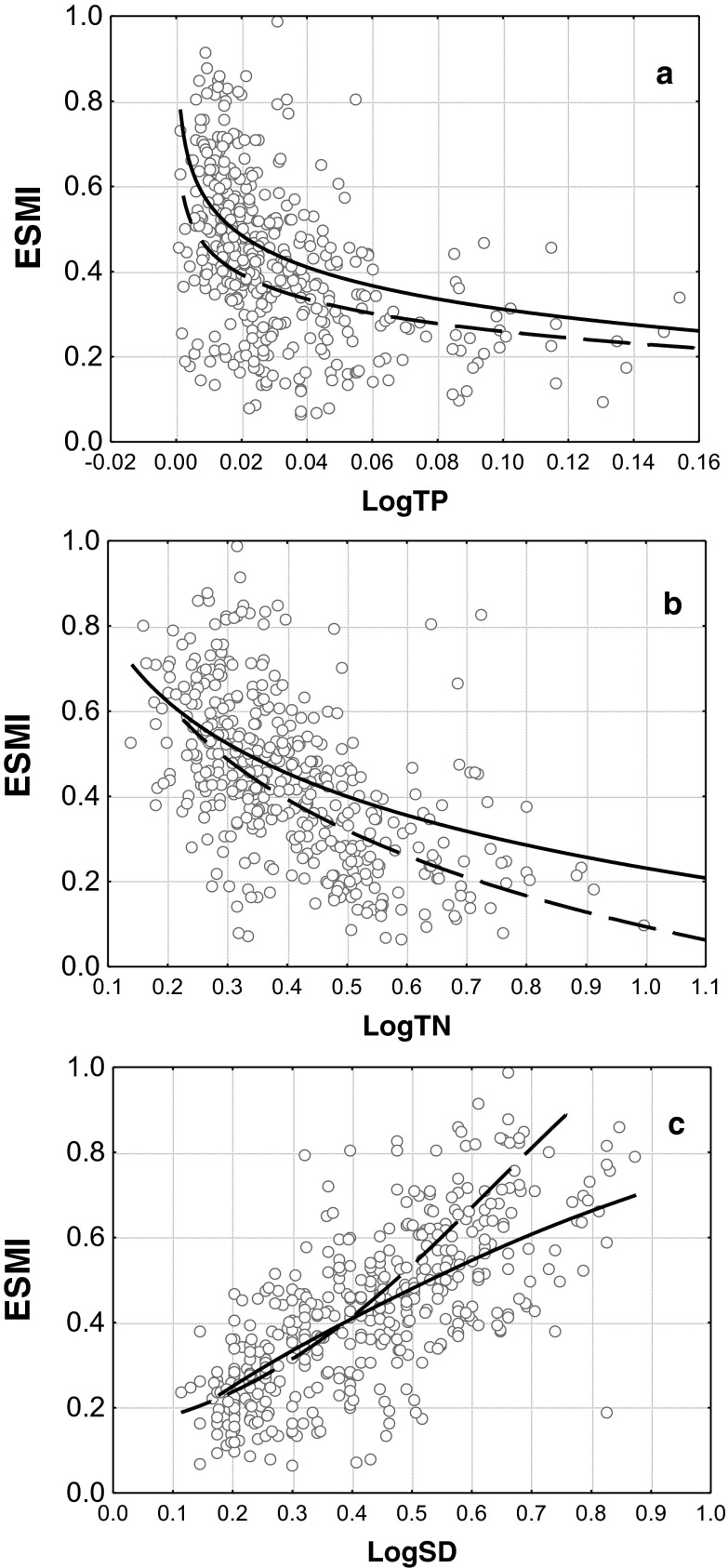
Relationships between ESMI and seasonal mean of TP (**a**), TN (**b**) and SD (**c**) in 427 lakes surveyed in the years 2007–2012. *TP*, *TN*, *SD* refer to the key in Table 1. *Lines* represent the logarithmic (**a**, **b**) or linear (**c**) model fit for response curve in stratified (*solid line*) and polymictic (*dashed line*) lakes

The ESMI method in the above presented form has been introduced into routine lake monitoring in Poland since 2007. After the first 6 years of use, the effectiveness of the ESMI index was validated based on biological and environmental data from 427 lakes surveyed in 2007–2012. A comparison of the validation results with the results obtained in 2006 revealed almost identical correlations between ESMI and water quality indicators in the evaluated period for stratified lakes and only slightly weaker correlations for polymictic lakes (Table 2). Furthermore, the pressure-response curves in stratified and polymictic lakes followed similar patterns (Fig. 4) and no significant differences in *R* values between relationships of stratified and polymictic lakes to TP (*p* = 0.09), TN (*p* = 0.89) and SD (*p* = 0.13) were found.

In the years 2009–2011, the method was subjected to the pan-European intercalibration process (Portielje et al. 2014), which revealed that the ESMI good/moderate class boundaries in both lake types were too lenient in comparison with classification systems of other Member States and had to be tightened. Moreover, as the ESMI index includes the typological factor N/P (Eq. 5) and it appeared to perform similarly in both mictic types (Table 2), there was no need to differentiate between stratified and polymictic lakes. Therefore, for all high-alkalinity stratified and polymictic lowland lakes, a single classification system was proposed, and class boundaries were modified (the *H*/*G* boundary reminded unmodified, G/M boundary was tightened to approximately 20 %, and the remaining boundaries adjusted accordingly; Table 4), to make the results of the ESMI-based assessment comparable with those of other European methods.

**Fig. 5:**
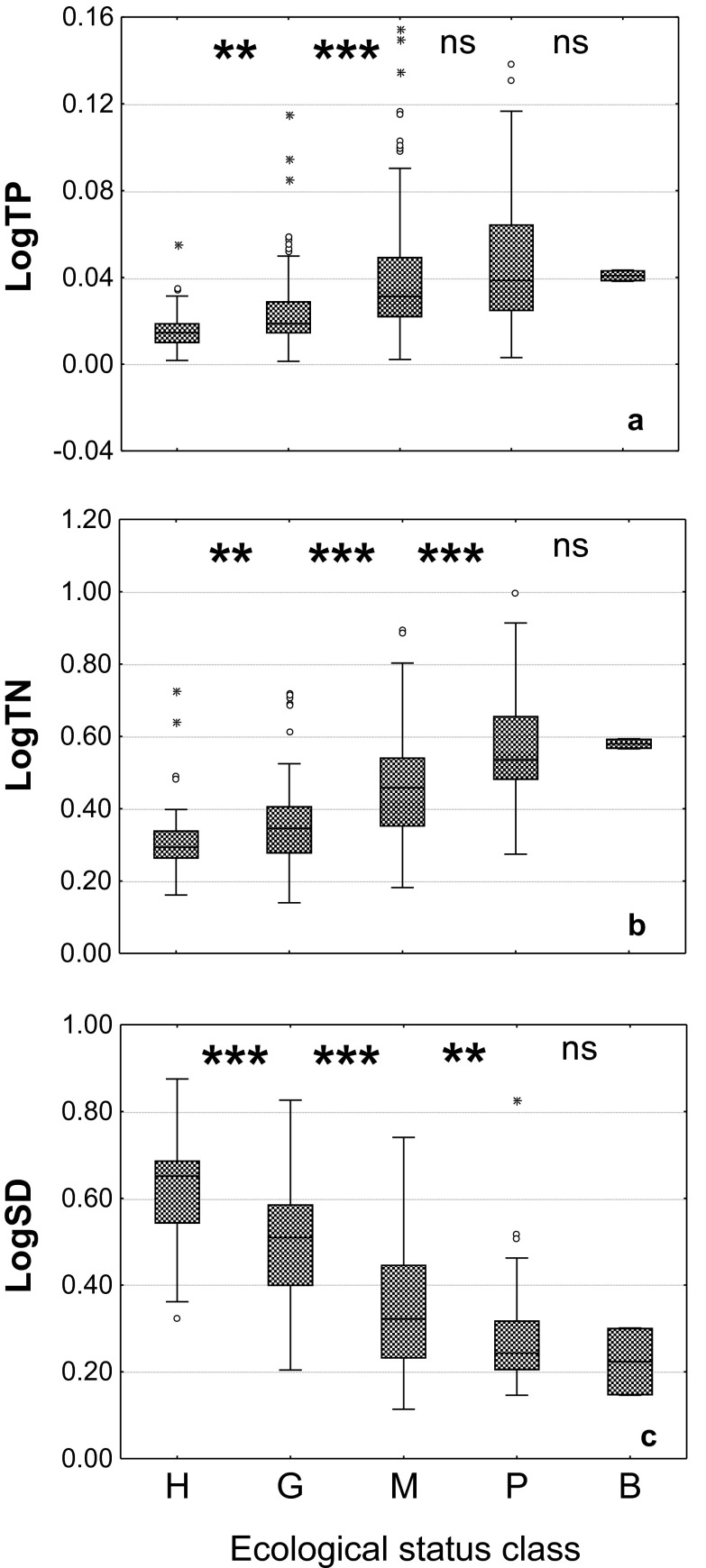
Distribution of TP (**a**), TN (**b**) and SD (**c**) in lakes classified to one of the five classes of ecological status according to the intercalibrated and harmonised class boundary values of the ESMI index. Boundary values as in Table 4. *Boxplots* 25-75^th^ percentiles with median, *whiskers* range, *circles* outliers, *stars* extreme values. *TP*, *TN*, *SD* refer to the key in Table 1; *H* high (*n* = 48), *G* good (*n* = 182), *M* moderate (*n* = 140), *P* poor (*n* = 55), *B* bad (*n* = 2) ecological status. *Stars* indicate the level of confidence in comparison of distribution of water quality parameters between subsequent classes of ecological status obtained in *t* test: ***p < 0.001, **p < 0.01, *p < 0.05, *ns* non-significant

In a pool of all the lakes in the monitoring database, including both stratified and polymictic lakes, ESMI correlated strongest with SD (*R* = 0.67), and significantly weaker with TN and TP (*R* = −0.56 and −0.43, respectively; Fig. 4). Although ESMI class boundaries were set regardless of water quality indicators, the distribution of eutrophication indicator values and means across ecological status classes differed significantly (Fig. 5), strongest for SD (ANOVA *F*_4;418_ = 79.01) and somewhat weaker for TN (*F*_4;421_ = 45.41) and TP (*F*_4;421_ = 20.27, all *p* < 0.0001). Moreover, the new classification provided a better discrimination of ecological status classes than originally (*F*_4;418_ = 60.10 for SD, *F*_4;421_ = 39.91 for TN and *F*_4;421_ = 20.37 for TP, all *p* < 0.0001 for original classification). For all the water quality indicators, the ESMI new classification differentiated best between three best classes (high, good and moderate), whereas in the worst status, a clear overlap between moderate and poor (TP), and poor and bad classes (TP, TN, SD) were observed (Fig. 5).

## Discussion

According to WFD requirements, the macrophyte-based assessment method should account for the taxonomic composition and abundance of the macrophyte community, produce numerical values that can be used to determine the Ecological Quality Ratio (EQR), account for natural variability in abiotic and biotic conditions of aquatic ecosystems (type-specific classification) and refer to natural conditions in undisturbed ecosystems (reference conditions).

The taxonomic composition of aquatic vegetation can be expressed by various numerical indicators, including the number of species/communities, the proportion between different species groups or indicators of taxonomic diversity. The absolute number of plant species or communities has limited relevance for evaluations of lake quality because it might be influenced by variations in the lake’s morphometric parameters such as size, length, bed slope, shoreline development, diversity of the bottom substrate, regardless of water quality (Duarte and Kalff 1986; Rørslett 1991; Vestergaard and Sand-Jensen 2000). The absolute number of species or communities does not exhibit a clear negative response to eutrophication pressure, as its response curve along the phosphorus gradient was found to be unimodal with the highest number of plant species found in habitats ranging from mesotrophic to eutrophic environments and the lowest in both nutrient-poor and nutrient-rich conditions (Rørslett 1991; Toivonen and Huttunen 1995; Murphy 2002; Penning et al. 2008). Quantitative ratios between functional species groups (sensitive to tolerant taxa) are used effectively in many European methods (Schaumburg et al. 2004; Penning et al. 2008; Sager and Lachavanne 2009), but are of limited value in studies of Polish lakes. Ecosystems with naturally eutrophic, calcium-rich waters are colonised mainly by eurytopic species with a relatively wide ecological amplitude and similar habitat requirements, but they are largely devoid of species that are particularly sensitive to eutrophication, which minimises the effectiveness of the above ratios.

In the ESMI method, the two biocenotic diversity indices, the actual one *H* and the maximum theoretically possible *H*_max_, create the taxonomic composition measure. Their ratio, which is an index of evenness *J*, expresses the structural simplification of plant systems resulting from anthropogenic pressure. In the ecological succession of natural non-disturbed ecosystems, the increase in taxonomic completeness leads to the achievement of the maximum species diversity (*H*_max_), with the minimum abundance of any particular species in the species pool (Akatov et al. 2009). The proportions of all taxa are balanced, whereas the phytocenotic diversity index *H* reaches high values and tends to *H*_max_. The value of *J* thus approximates to 1. When the phytocenotic balance is disturbed, for example, by anthropogenic pressure, vegetation patterns are simplified, some communities disappear, whereas others become prevalent, and the values of *H* and *J* decrease (Rejewski 1981; Ciecierska et al. 2010).

The biocenotic diversity index *H*, estimates biological variability and is strongly affected by the number of species (or communities in the case of ESMI), whereas *J* = *H*/log(*S*) is a normalisation of *H* and quantifies the distribution of *S* across the community. Although Pielou’s index has been criticised for its low effectiveness and frequent misinterpretation errors (Beisel and Moreteau 1997; Heip et al. 1998; Jost 2010), it has been argued to be an excellent measure of relative evenness (Jost 2010), and continues to be one of the most widely used evenness indicators in ecological studies.

The abundance of aquatic vegetation is usually expressed as macrophyte coverage and/or maximum colonisation depth (Søndergaard et al. 2013). The total vegetated area, i.e. the area of the phytolittoral, is generally taken into account. This metric can be expressed in absolute units or in terms of the percentage of lake area; therefore, it might be relatively unreliable, as it is determined by the lake’s morphometric parameters rather than its ecological status. In the ESMI method, the colonisation index (*Z*) i.e., the ratio of lake area occupied by macrophytes to the area potentially available to plants, was applied. It has been assumed that in a lake characterised by at least a good ecological status, the phytolittoral surface area should not be less than the area bound by the 2.5 isobath (Rejewski 1981; Ciecierska 2008) which corresponds to a colonisation depth of up to 2.5 m. A similar macrophyte colonisation depth range of 3.0 m (for stratified lakes) to 2.3 m (for polymictic lakes; 2.65 m on average) was suggested by Poikane et al. (2014) as a good/moderate ecological status boundary value.

The value of the colonisation index increases with the maximum colonisation depth. In theory, under reference conditions where the average colonisation depth is 5.0 m in deep lakes and 3.8 m in shallow lakes (Table 3), the value of *Z* reaches a minimum of 1.5–2.0, and it approximates to 1 in lakes with a good ecological status and declines radically in more degraded ecosystems. Although the colonisation index is strongly correlated with the maximum colonisation depth, it is also used to determine vegetation cover density. Two lakes, where one is characterised by very dense vegetation cover and the other by sparse cover, might represent different conditions, even if they have an identical maximum colonisation depth. In general, the higher the colonisation index is, the more abundant is the vegetation cover. The above and also former results by Ciecierska (2008), Ciecierska et al. (2010) and Kolada (2010) clearly indicate that index *Z* is a highly useful tool in assessments of the ecological status of lakes.

**Fig. 6:**
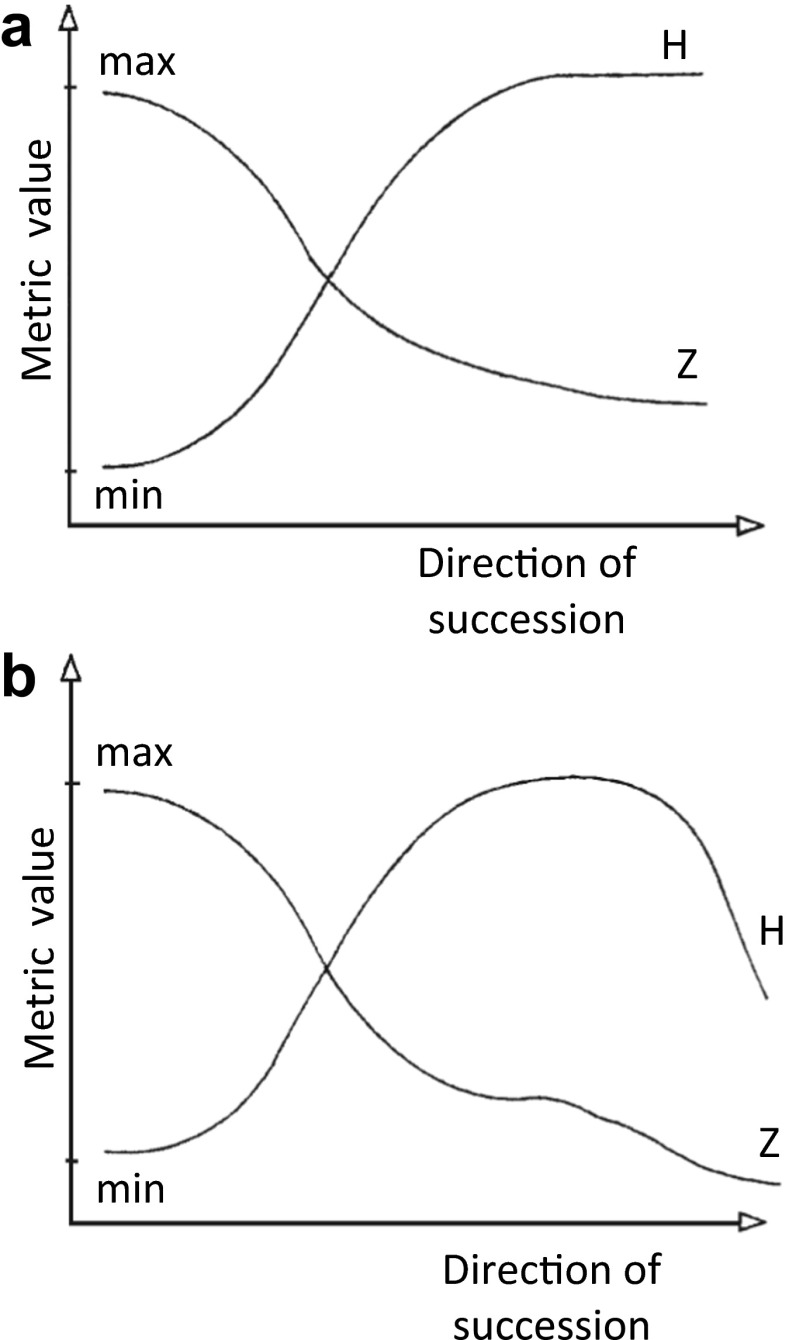
Theoretical distribution of the colonisation index (*Z*) and the biocenotic diversity index (*H*) in lake ecosystems subjected to hypothetical autogenic succession (**a**) and actual anthropogenic succession (**b**) (reprint from Rejewski 1981, modified)

The two discussed ESMI components, diversity index *H* and abundance index *Z*, are negatively correlated. Together, they form a system of coordinates that denotes the position of a lake on a curve, which depicts the course of succession (Rejewski 1981, Fig. 6). Theoretically, in ecosystems characterised by a certain degree of ecological resistance, vegetation patterns tend to equilibrium (climax), and *H* and *Z* tend towards stable values that correspond to the capacity of a habitat (Fig. 6a). In practice, in ecosystems affected by disturbances of a sufficient magnitude or duration, the value of *H* decreases when the ecological threshold is exceeded (Fig. 6b). In most degraded ecosystems in near-climax communities, a decrease in *Z* values becomes accompanied by a decrease in *H* values.

The combination of *H* and *Z* was demonstrated to work effectively, and the ESMI multimetric responded adequately across the eutrophication gradient at the initial stages of index development (in 165 lake-years used in 2006 to develop the method) and during successive evaluation (in 427 lakes surveyed in 2007–2013; Table 2, Fig. 4). The slightly weaker correlation between ESMI and water quality indicators in 2013 than 2006 might have resulted from the low quality of at least some biological data (monitoring data, not research data) or from a higher sampling frequency in the redesigned monitoring regime (three instead of two times a year). In our study, a considerably stronger relationship between ESMI and SD than between ESMI and TP or TN was found (Table 2). Light is crucial for photosynthesis and its availability is a primary factor that determines the occurrence of aquatic plants (Middleboe and Markager 1997; van den Berg et al. 2003; Lacoul and Freedman 2006). The increase in phosphorus and nitrogen concentrations promotes the intensive growth of suspended algae and a decrease in light attenuation. As a result of light climate deterioration along with a progressive eutrophication process, plants occupying the deepest zones of the phytolittoral tend to retreat to shallower waters, the maximum colonisation depth decreases, sensitive taxa are replaced by more tolerant ones, up to the complete extinction of submerged vegetation, and the domination of rush communities (Middleboe and Markager 1997; Schwarz et al. 2002; Squires et al. 2002; van den Berg et al. 2003; Kolada 2014). Hence, water turbidity (usually related to Chl*a* concentration) has a direct impact on the composition, distribution and abundance of macrophytes, whereas the effect of nutrients is indirect via its significant relationship to most trophic levels (Jeppesen et al. 2000; Søndergaard et al. 2010). Therefore, the stronger response of macrophytes to water visibility (*R* = 0.67) than to nutrient concentrations (*R* = −0.56 for TN and −0.43 for TP) observed in our study is not surprising.

For the regression models used in aquatic sciences, Prairie (1996) suggested *R* ^*2*^ ≥ 0.65 as a meaningful threshold for biological responses that provide a suitable resolution power for distinguishing between at least two classes. In practice, the predictive power of most of the existing macrophyte indices is far away from this threshold (Prairie 1996; Demars et al. 2012). The physical and chemical properties of water are characterised by relative instability, and the results of individual studies are rarely used to determine the floristic and spatial structure of aquatic vegetation. Most attempts to correlate physical and chemical parameters with a given species or community have failed to produce satisfactory results (Rejewski 1981). Considering that the responses of biological assemblages to pressure are usually non-linear and are determined by a large variation in unpredictable factors, the correlations demonstrated in our study were assumed to be satisfactory. The reliability of ESMI as a lake ecological status indicator has also been confirmed within the intercalibration process, as the metric performed equally well as the other EU methods (Portielje et al. 2014).

The assessment and classification of the ecological status of water bodies should refer to type-specific reference conditions. In the original ESMI classification, two types of Polish lowland lakes were distinguished (Table 4). According to the abiotic typology scheme for Polish lakes, as many as 13 lake types have been identified (Kolada et al. 2005). It has been demonstrated however, that in Central European lowlands, the actual lake vegetation patterns are less diverse than the theoretical patterns expected based on variations in environmental conditions. In practice, hard-water lowland lakes represent a single macrophyte-type with a predominance of stonewort communities in the reference state (*Chara*-type; Schaumburg et al. 2004; Free et al. 2007; Willby et al. 2009; Kolada 2010; Azzella et al. 2013a). These findings are consistent with the results of our study. Due to differences in the macrophyte dominance structure, stratified and non-stratified lakes are usually analysed separately (Schaumburg et al. 2004; Free et al. 2007; Willby et al. 2009), which was also the case with the ESMI method in its original version (Table 4). However, no differences in the distribution of ESMI between stratified and polymictic lakes either in reference (Fig. 2, Table 3) or in non-reference sites (Table 3) were observed. Comparing stratified and polymictic lakes, the strength of the relationships between ESMI and water quality indicators were not statistically different. Therefore, the decision to develop one classification system for all hard-water lowland lakes, as one outcome of the intercalibration process, appears fully justified.

The ESMI follows a negative logarithmic function; thus, the original classification system was based on a log-divided boundary scale. The resulting quality classes were wide at the high/good end and narrowed down towards the lowest index values (Table 4). The metric is most sensitive to changes at high values and becomes increasingly less sensitive as the ecological status declines. ESMI behaves inversely compared with most other logarithm-based biotic indices, including, e.g. the Shannon-Wiener diversity index *H*, whose pressure-response curves progressively flatten towards the quasi-asymptotic maximum value. Their use in bioassessment is hence questionable, as they fail to detect even large changes at high index values (Miccoli et al. 2013). Conversely, ESMI might serve as a sentinel index in the good/high end of the metric spectrum, as it can detect small changes in the macrophyte community at the beginning of the environmental problem (best discrimination between high, good and moderate status, Fig. 5). The poor discrimination between the poor and bad status class in our study was probably caused by an insufficient number of lakes being assessed as bad (*n* = 2). The main objection to the original ESMI classification derived from the intercalibration exercise concerned too lenient values of the good/moderate boundary and too broad range of the good status class (a large fraction of lakes falling into the good class). The tightening of the original ESMI class boundaries improved the discrimination of ecological status classes and was a mandatory process to provide comparability of the ESMI-based assessment results with the outcomes of other macrophyte-based methods for evaluating the ecological status of lakes in Central European lowlands.

The sampling method designed by us for routine lake monitoring in Poland is based on a transect survey. This technique is recommended in many EU countries (Kolada et al. 2009) as a cost-effective procedure and has been demonstrated to provide reliable results at a reasonable sampling effort (Schaumburg et al. 2004; Pall and Moser 2009; Azzella et al. 2013b; Dudley et al. 2013; Kanninen et al. 2013).

## Conclusions

The ESMI evaluates the taxonomic composition and abundance of macrophyte communities. The index values range from 0 to 1, where 1 denotes the reference conditions, and values decrease as the quality of the ecosystem deteriorates. The ESMI responds satisfactorily to eutrophication pressure. Although only a single ESMI-based classification system has been developed for high-alkalinity lowland lakes, the variations in vegetation patterns in different abiotic lake types had been previously recognised. Therefore, the ESMI fulfils all of the requirements set by the Water Framework Directive for biological indicators for the assessment and classification of the ecological status of water bodies.

## Electronic supplementary material

Below is the link to the electronic supplementary material.

ESM 1 (PDF 182 kb)

ESM 2 (PDF 205 kb)
