# Supplementary material for: ESMI: a macrophyte index for assessing the ecological status of lakes
Source: Environ Monit Assess. 2014 May 18;186(9):5501–17. doi: 10.1007/s10661-014-3799-1 (PMC4112054; doi:10.1007/s10661-014-3799-1)
Supplement: Supplementary file 1 — (PDF 182 kb) [file 10661_2014_3799_MOESM1_ESM.pdf]

Environmental Monitoring and Assessment

Hanna Ciecierska[1], Agnieszka Kolada[2]

ESMI: a Macrophyte Index for assessing the Ecological Status of lakes

[1] University of Warmia and Mazury, Department of Botany and Nature Protection, Plac Łódzki 1, 10-727 Olsztyn-Kortowo, Poland, e-mail: makrof@uwm.edu.pl; [2] Institute of Environmental Protection-National Research Institute, Department of Freshwater Assessment Methods and Monitoring, Kolektorska 4, 01-692 Warszawa, e-mail: akolada@ios.edu.pl

Appendix 1 The list of lakes used in the study; lakes ordered alphabetically and then chronologically. Mixing type: S – stratified, P – polymictic; ecological status according to ESMI (original classification): REF – reference lake, H – high, G – good, M – moderate, P – poor, B – bad; n.d. – no data available

No.	Lake name	Year of macrophyte survey	Source of macrophyte data	Year of water sampling	Area (km^2)	Mean depth (m)	Mixing type	ESMI	Ecol. status
1	Bachotek	2005	Jusik and Zgoła 2005	2004	2.11	7.2	S	0.590	G
2	Bełdany	2001	Ciecierska 2008	2004	9.41	10.0	S	0.164	P
3	Będgoszcz	2003	Ciecierska 2008	2003	2.64	5.5	P	0.097	P
4	Białe Gostynińskie	2006	Kolada 2008	2005	1.50	9.9	S	0.630	REF
5	Białe Wigierskie	1998	Ciecierska 2008	n.d.	0.99	13.2	S	0.807	REF
6	Białoławki	2001	Ciecierska 2008	2002	2.11	9.8	S	0.480	G
7	Borzymowskie	1986	Samosiej 1987	n.d.	1.75	4.2	P	0.365	G
8	Brożane	2005	Kolada 2008	2004	0.45	6.7	S	0.102	P
9	Budziszewskie	2006	Ciecierska 2008	2003	1.63	5.0	S	0.173	M
10	Busznica	2003	Ciecierska 2008	2003	0.49	6.8	S	0.602	REF
11	Chodeckie	1986	Samosiej 1987	n.d.	0.40	5.5	S	0.329	M
12	Ciche	2005	Jusik and Zgoła 2005	2003	1.11	8.8	S	0.706	G
13	Ciechomickie	2006	Kolada 2008	2005	0.47	4.9	S	0.436	G
14	Dębno	2005	Jusik and Zgoła 2005	2002	0.60	5.5	S	0.768	G
15	Długie Leśne	1981	Rejewski 1981	n.d.	0.40	1.2	P	0.765	G
16	Długie Olsztyńskie	1989	Ciecierska 2008	n.d.	0.27	5.3	S	0.043	B
17	Długie Olsztyńskie	2005	Ciecierska 2008	n.d.	0.27	5.3	S	0.361	M
18	Długie Polne	1986	Samosiej 1987	n.d.	0.89	n.d.	P	0.382	G
19	Długie Szczycieńskie	1989	Ciecierska 2008	n.d.	0.62	2.3	P	0.191	M
20	Długie Wigierskie	1998	Ciecierska 2008	2003	0.80	6.4	S	0.665	REF
21	Duś	1996	Ciecierska 2008	n.d.	0.36	2.8	P	0.914	REF
22	Foluskie	2006	Ciecierska 2008	2005	0.62	10.0	S	0.491	G
23	Garbaś	1981	Endler et al. 1989a	n.d.	0.43	10.2	S	0.677	H
24	Garbaś	2006	Kolada 2008	n.d.	0.43	10.2	S	0.733	H
25	Gardliczno Duże	1981	Rejewski 1981	n.d.	0.32	7.5	S	0.366	G
26	Gardliczno Duże	2006	Kolada 2008	n.d.	0.32	7.5	S	0.075	B
27	Gardyńskie	1997	Ciecierska 2008	n.d.	0.83	2.5	P	0.695	G
28	Głuche Duże	1981	Rejewski 1981	n.d.	0.45	8.0	S	0.812	G

No.	Lake name	Year of macrophyte survey	Source of macrophyte data	Year of water sampling	Area (km^2)	Mean depth (m)	Mixing type	ESMI	Ecol. status
29	Górskie	2006	Kolada 2008	n.d.	0.45	3.2	S	0.646	H
30	Guzianka Duża	1997	Ciecierska 2008	1998	0.60	6.5	S	0.233	M
31	Guzianka Mała	1997	Ciecierska 2008	n.d.	0.37	2.7	S	0.421	G
32	Gwiazdy	2005	Kolada 2008	2001	2.10	14.0	S	0.557	REF
33	Iłgielk	2001	Ciecierska 2008	n.d.	0.34	2.8	P	0.035	B
34	Inulec	1995	Ciecierska 2008	n.d.	1.78	4.6	S	0.565	G
35	Jakuba	1989	Ciecierska 2008	n.d.	0.23	2.7	P	0.093	P
36	Jasień Południowy	2006	Kolada 2008	2006	3.37	7.5	S	0.251	M
37	Jasień Północny	2006	Kolada 2008	2006	2.41	9.1	S	0.355	G
38	Jegocin Wielki	1997	Ciecierska 2008	1998	1.27	9.0	S	0.751	REF
39	Jegocin Wielki	2004	Ciecierska 2008	2004	1.27	9.0	S	0.783	REF
40	Jegocinek	1997	Ciecierska 2008	1996	0.52	7.1	S	0.218	B
41	Jemiołowo	1989	Ciecierska 2008	n.d.	0.49	2.8	S	0.451	G
42	Jeziorak Mały	1991	Ciecierska 2008	n.d.	0.26	3.4	P	0.027	B
43	Jeziorak Mały	2000	Ciecierska 2008	n.d.	0.26	3.4	P	0.063	P
44	Kajkowskie	1986	Ciecierska 2008	n.d.	0.29	3.8	S	0.341	G
45	Kamieniczno	2005	Kolada 2008	2000	1.21	9.6	S	0.482	H
46	Karaśnia	1986	Samosiej 1987	n.d.	0.43	n.d.	P	0.483	G
47	Kęty	1996	Ciecierska 2008	n.d.	0.97	5.7	S	0.617	G
48	Kiełbonki	1998	Ciecierska 2008	n.d.	0.30	2.2	P	0.960	H
49	Kierwik	1997	Ciecierska 2008	n.d.	0.60	6.1	S	0.302	M
50	Kierzkowskie	2006	Ciecierska 2008	2005	0.77	8.0	S	0.392	G
51	Kołowin	1986	Środa 1990	1989	0.78	4.0	P	0.770	REF
52	Kołowin	1996	Ciecierska 2008	n.d.	0.78	4.0	P	0.509	G
53	Kołowin	1998	Ciecierska 2008	n.d.	0.78	4.0	P	0.585	G
54	Kołowin	1999	Ciecierska 2008	2004	0.78	4.0	P	0.510	G
55	Kołowin	2000	Ciecierska 2008	2004	0.78	4.0	P	0.583	REF
56	Kołowin	2003	Ciecierska 2008	2004	0.78	4.0	P	0.561	REF
57	Kortowskie	1990	Ciecierska 2008	n.d.	0.90	5.9	S	0.235	M
58	Kortrowskie	2001	Ciecierska 2008	n.d.	0.90	5.9	S	0.177	M
59	Kraksy Duże	1989	Ciecierska 2008	n.d.	0.44	1.1	P	0.579	G
60	Krępsko Długie	2005	Kolada 2008	2005	0.74	7.6	S	0.447	REF
61	Kromszewickie	1986	Samosiej 1987	n.d.	0.89	8.6	S	0.380	G
62	Królewskie (Łokacz)	2005	Jusik and Zgoła 2005	2005	0.51	1.8	P	0.113	M
63	Krusznik	1998	Ciecierska 2008	n.d.	0.27	3.5	S	0.850	H
64	Krutyńskie	1996	Ciecierska 2008	n.d.	0.55	1.7	P	0.886	REF
65	Krzywe (Ukiel)	2000	Ciecierska 2008	2004	4.12	10.6	S	0.575	G
66	Księże	1981	Rejewski 1981	n.d.	0.75	2.7	P	0.747	G
67	Księże	2006	Kolada 2008	2003	0.75	2.7	P	0.092	P
68	Kuc	1995	Ciecierska 2008	n.d.	0.99	8.0	S	0.931	REF
69	Kuc	2003	Ciecierska 2008	n.d.	0.99	8.0	S	0.876	REF
70	Laska	1981	Rejewski 1981	n.d.	0.70	1.4	P	0.911	H
71	Laska	2006	Kolada 2008	2003	0.70	1.4	P	0.221	M
72	Leszczewek	1998	Ciecierska 2008	n.d.	0.21	3.6	P	0.346	G
73	Lubieńskie	1986	Samosiej 1987	n.d.	0.89	3.9	S	0.207	M
74	Lucieńskie	2006	Kolada 2008	2004	2.01	8.3	S	0.541	G
75	Łabędź	2001	Ciecierska 2008	2001	3.08	3.7	P	0.261	M
76	Łagowskie	2006	Kolada 2008	2005	0.82	5.3	S	0.327	G
77	Łąckie Duże	2006	Kolada 2008	2005	0.56	3.0	P	0.065	B
78	Łąckie Małe	2006	Kolada 2008	2005	0.27	1.8	P	0.153	B
79	Łękuk Wielki	1983	Endler et al. 1989b	n.d.	0.21	5.2	S	0.529	G
80	Łękuk Wielki	1998	Grzybowski et al. 2000	n.d.	0.21	5.2	S	0.392	G

No.	Lake name	Year of macrophyte survey	Source of macrophyte data	Year of water sampling	Area (km^2)	Mean depth (m)	Mixing type	ESMI	Ecol. status
81	Łękuk Wielki	2003	Ciecierska 2008	n.d.	0.21	5.2	S	0.601	G
82	Łętowskie	2003	Ciecierska 2008	2003	4.02	8.2	P	0.430	G
83	Łuknajno	1970	Polakowski et al. 1973	n.d.	6.80	0.6	P	0.737	H
84	Łuknajno	1993	Królikowska 1997	n.d.	6.80	0.6	P	0.505	H
85	Łuknajno	2003	Ciecierska 2008	n.d.	6.80	0.6	P	0.773	REF
86	Majcz Mały	2001	Ciecierska 2008	n.d.	0.20	1.8	P	0.939	REF
87	Majcz Wielki	1995	Ciecierska 2008	1996	1.64	6.0	S	0.751	REF
88	Majcz Wielki	1998	Ciecierska 2008	1996	1.64	6.0	S	0.781	REF
89	Majcz Wielki	2000	Ciecierska 2008	2004	1.64	6.0	S	0.787	REF
90	Majcz Wielki	2004	Ciecierska 2008	2004	1.64	6.0	S	0.801	REF
91	Marksoby	2006	Ciecierska 2008	n.d.	1.55	4.5	S	0.754	H
92	Miedwie	2005	Ciecierska 2008	2005	35.27	19.3	S	0.553	H
93	Mikołajskie	1997	Ciecierska 2008	1997	4.98	11.2	S	0.104	P
94	Milachowo	1981	Rejewski 1981	n.d.	0.65	1.9	P	0.467	G
95	Modzerowskie	1986	Samosiej 1987	n.d.	1.46	3.3	P	0.443	G
96	Mojtyny	1997	Ciecierska 2008	n.d.	0.28	3.5	P	0.925	H
97	Mojtyny	2003	Ciecierska 2008	n.d.	0.28	3.5	P	0.872	H
98	Mokre	1996	Ciecierska 2008	1996	8.41	12.7	S	0.498	G
99	Mokre	2004	Ciecierska 2008	2004	8.41	12.7	S	0.617	G
100	Morzycko	2004	Ciecierska 2008	2004	3.43	14.5	S	0.634	H
101	Muliczne	1998	Ciecierska 2008	2003	0.26	4.7	S	0.755	H
102	Nawiadki	1996	Ciecierska 2008	n.d.	0.26	4.2	P	0.799	H
103	Nawiady	1997	Ciecierska 2008	1992	2.00	9.0	S	0.628	REF
104	Nobliny (Niewlino)	2002	Ciecierska 2008	2002	1.53	6.1	S	0.585	H
105	Nowowiejskie	2005	Jusik and Zgoła 2005	2002	0.29	1.2	P	0.067	P
106	Ostrowieckie	2006	Ciecierska 2008	2005	1.60	6.0	S	0.431	G
107	Ostrowo	2003	Ciecierska 2008	2003	3.78	2.7	P	0.270	G
108	Parszczenica	1981	Rejewski 1981	n.d.	0.78	1.8	P	0.880	G
109	Parszczenica	2006	Kolada 2008	2003	0.78	1.8	P	0.127	M
110	Partęczyny Wielkie	2005	Jusik and Zgoła 2005	2003	3.24	6.8	S	0.646	G
111	Parzyn	1981	Rejewski 1981	n.d.	0.53	1.0	P	0.703	H
112	Piaseczno	2005	Ciecierska 2008	2005	0.54	4.5	S	0.341	G
113	Piaski	2003	Ciecierska 2008	2003	0.92	2.1	P	0.171	M
114	Pierty	1998	Ciecierska 2008	n.d.	2.28	10.4	S	0.643	G
115	Piłwąg	2005	Kolada 2008	n.d.	1.35	1.5	P	0.077	P
116	Płaskie	2005	Kolada 2008	n.d.	0.57	5.0	S	0.031	B
117	Płęsno	1981	Rejewski 1981	n.d.	0.95	16.0	S	0.738	G
118	Płęsno	2006	Kolada 2008	n.d.	0.95	16.0	S	0.162	P
119	Płoń	2003	Ciecierska 2008	2003	7.91	2.8	P	0.053	P
120	Popielewskie	2006	Ciecierska 2008	2003	3.00	12.0	S	0.175	M
121	Probarskie	1995	Ciecierska 2008	n.d.	2.01	9.2	S	0.733	G
122	Probarskie	2004	Ciecierska 2008	2004	2.01	9.2	S	0.717	G
123	Przedeckie (Przedecz)	1986	Samosiej 1987	n.d.	0.88	1.0	P	0.182	B
124	Przytoczno	2006	Ciecierska 2008	n.d.	2.28	5.0	S	0.086	B
125	Radomno	2006	Ciecierska 2008	2005	0.99	3.0	S	0.221	M
126	Redykajny	2001	Ciecierska 2008	n.d.	0.30	8.0	S	0.847	H
127	Robotno	2005	Jusik and Zgoła 2005	n.d.	0.50	5.9	S	0.607	G
128	Sasek Mały (Szoby)	2006	Ciecierska 2008	2006	3.19	2.0	P	0.526	G
129	Skanda	1989	Dziedzic 2002	n.d.	0.51	5.8	S	0.177	M
130	Skarp	1998	Ciecierska 2008	n.d.	0.23	5.7	P	0.862	H
131	Sosno	2005	Jusik and Zgoła 2005	2003	1.88	5.0	S	0.523	G
132	Strażym	2005	Jusik and Zgoła 2005	n.d.	0.73	3.5	P	0.256	M

No.	Lake name	Year of macrophyte survey	Source of macrophyte data	Year of water sampling	Area (km^2)	Mean depth (m)	Mixing type	ESMI	Ecol. status
133	Strzeszowskie	2002	Ciecierska 2008	2002	1.27	7.5	S	0.311	M
134	Sukiel (Likuskie)	1989	Ciecierska 2008	n.d.	0.21	6.6	S	0.535	G
135	Suskie	2000	Ciecierska 2008	2004	0.63	2.3	P	0.225	M
136	Szwałk Mały	2005	Kolada 2008	n.d.	0.70	4.3	P	0.545	G
137	Śluza	1981	Rejewski 1981	n.d.	0.76	3.1	P	0.762	G
138	Tarnowskie Wielkie	2006	Kolada 2008	2006	0.92	3.8	P	0.523	G
139	Trackie	1988	Ciecierska 2008	n.d.	0.53	2.1	P	0.175	M
140	Trześniowskie (Ciecz)	2006	Kolada 2008	2005	1.86	19.3	S	0.326	G
141	Tuchlin	1998	Ciecierska 2008	n.d.	2.19	2.9	P	0.596	G
142	Tyrkło	1998	Ciecierska 2008	1996	2.36	9.7	S	0.285	M
143	Uplik	1996	Ciecierska 2008	n.d.	0.61	2.8	P	0.377	G
144	Warszyn	1981	Rejewski 1981	n.d.	0.35	2.6	P	0.505	G
145	Wądół	2006	Ciecierska 2008	n.d.	1.55	5.0	S	0.362	G
146	Wągiel Duży	1997	Ciecierska 2008	1999	1.50	4.2	S	0.206	M
147	Wągiel Mały	1997	Ciecierska 2008	n.d.	0.27	2.3	P	0.175	M
148	Wejsuny	1998	Ciecierska 2008	n.d.	0.38	3.6	P	0.601	G
149	Wełtyńskie	2002	Ciecierska 2008	2002	3.10	4.1	P	0.712	REF
150	Wielimie	2003	Ciecierska 2008	2003	17.55	2.2	P	0.065	P
151	Wierzbowskie	1995	Ciecierska 2008	n.d.	1.04	4.8	S	0.559	G
152	Wigryny	1998	Ciecierska 2008	n.d.	0.25	1.7	P	0.678	G
153	Wilkowskie	2006	Kolada 2008	2004	1.31	8.9	S	0.476	G
154	Wiłkokuk	2005	Kolada 2008	n.d.	0.39	3.8	S	0.680	REF
155	Witoczno	1981	Rejewski 1981	n.d.	1.01	4.3	P	0.572	G
156	Wolickie	2006	Ciecierska 2008	2005	2.44	5.0	S	0.264	M
157	Wukśniki	2005	Ciecierska 2008	2005	1.17	23.3	S	0.807	REF
158	Załom Wielki	2006	Ciecierska 2008	n.d.	1.05	5.0	S	0.417	G
159	Zamieć (Szczuczarz)	2006	Ciecierska 2008	n.d.	1.38	6.0	S	0.719	H
160	Zbąszyńskie (Błędno)	2005	Jusik and Zgoła 2005	2002	7.43	3.5	P	0.189	M
161	Zbiczno	2005	Jusik and Zgoła 2005	2004	1.29	11.7	S	0.241	M
162	Zdrużno	1997	Ciecierska 2008	2001	2.50	5.4	S	0.580	G
163	Zdworskie	2006	Kolada 2008	2005	3.55	2.1	P	0.125	M
164	Zelwa	2005	Kolada 2008	2001	1.04	5.8	S	0.814	REF
165	Żelewo	2004	Ciecierska 2008	2004	0.68	3.7	P	0.258	M

References

Ciecierska, H. (2008). Macrophyte based indices of the ecological state of lakes [Makrofity jako wskaźniki stanu ekologicznego jezior]. Dissertations and Monographs 139, University of Warmia and Mazury in Olsztyn (in Polish with English summary).

Dziedzic, J. (2002). Changes of vegetation in Lake Kortowskie [Zmiany roślinności jeziora Kortowskiego]. University of Olsztyn, Olsztyn (manuscript in Polish).

Endler, Z., Dziedzic, J., & Pietraszewski, W. (1989a). Actual vegetation in the catchment of lakes Stopka and Garbaś Duży in Borecka Forest [Roślinność rzeczywista zlewni jezior Stopka i Garbaś Duży w Puszczy Boreckiej]. Acta Academiae Agriculturae ac Technicae Olstenensis. Agricultura, 49, 3–11 (in Polish with English summary).

Endler, Z., Dziedzic, J., & Pietraszewski, W. (1989b). Actual vegetation in the catchment of Lake Łękuk Wielki in Borecka Forest [Roślinność rzeczywista zlewni jeziora Lękuk Wielki w Puszczy Boreckiej]. Acta Academiae Agriculturae ac Technicae Olstenensis. Agricultura, 49, 13–20 (in Polish with English summary).

Grzybowski, M., Endler, Z., & Juśkiewicz, B. (2000). Plant cover of Lake Łękuk Wielki located in the Borecka Primeval Forest. Natural Sciences 4, 183–197.

Jusik, Sz., Zgoła, T. (2005). The characteristic of macrophytes of lakes In Brodnicki Landscape Park [Opracowanie charakterystyki makrofitów w jeziorach Brodnickiego Parku Krajobrazowego]. Agriculture University in Poznań, Poznań (manuscript in Polish).

Kolada, A. (2008). The diversity of aquatic vegetation in selected Polish lowland lake types under the influence of anthropogenic pressure. PhD Theses. Adam Mickiewicz University in Poznań, Poznań (manuscript in Polish).

Królikowska, J. (1997). Eutrophication processes in a shallow, macrophyte-dominated lake - species differentiation, biomass and the distribution of submerged macrophytes in Lake Łuknajno (Poland). Hydrobiologia, 342/343, 411–416.

Polakowski, B., Dziedzic, J., & Polakowska, E. (1973). Vegetation of nature reserve „Jezioro Łuknajno" in Mazurian Lakeland [Roślinność rezerwatu "Jezioro Łukniany" na Pojezierzu Mazurskim]. Ochrona Przyrody (Nature Protection), 38, 85–114 (in Polish).

Rejewski, M. (1981). Lake vegetation of the Laska region in the Tuchola Forests [Roślinność jezior rejonu Laski w Borach Tucholskich]. Treatises of Mikołaj Kopernik University in Toruń (in Polish with English summary).

Samosiej, L. (1987). The effect of anthropopressure on littoral vegetation of lakes in agriculrtural area of Southern Kujawy [Wpływ antropopresji na roślinność litoralu jezior w krajobrazie rolniczym południowych Kujaw]. PhD Theses. University in Łódź. Łódź (manuscript in Polish).

Środa, M. (1990). Vegetation of Lake Kolowin in Mrągowo Lakeland [Szata roślinna jeziora Kołowin na Pojezierzu Mrągowskim]. Acta Academiae Agriculturae ac Technicae Olstenensis, Protectio Aquarum et Piscatoria, 18, 19–25 (in Polish with English summary).
