# Supplementary material for: ESMI: a macrophyte index for assessing the ecological status of lakes
Source: Environ Monit Assess. 2014 May 18;186(9):5501–17. doi: 10.1007/s10661-014-3799-1 (PMC4112054; doi:10.1007/s10661-014-3799-1)
Supplement: Supplementary file 2 — (PDF 205 kb) [file 10661_2014_3799_MOESM2_ESM.pdf]

Environmental Monitoring and Assessment

Hanna Ciecierska[1], Agnieszka Kolada[2]

ESMI: a Macrophyte Index for assessing the Ecological Status of lakes

[1] University of Warmia and Mazury, Department of Botany and Nature Protection, Plac Łódzki 1, 10-727 Olsztyn-Kortowo, Poland, e-mail: makrof@uwm.edu.pl; [2] Institute of Environmental Protection-National Research Institute, Department of Freshwater Assessment Methods and Monitoring, Kolektorska 4, 01-692 Warszawa, e-mail: akolada@ios.edu.pl

Appendix 2 The list of the plant communities of aquatic, rush and sedge rush vegetation identified in the phytolittoral of 165 Polish lowland lakes (research data from the period 1970-2006) which were used to develop the Ecological State Macrophyte Index (ESMI). Data for reference and non-reference lakes presented separately. The synaxonomic system according to Brzeg and Wojterska (2001) and Matuszkiewicz (2002) was applied. Ass. – association, f. – form, Ass. – association

Plant communities	Reference lakes (n=26)			Non-reference lakes (n=139)		
	No of occurrence	Frequency (% of lakes)	Mean cover (%)	No of occurrence	Frequency (% of lakes)	Mean cover (%)
Class: **Lemnetea**						
Lemno-Utricularietum vulgaris	4	15.4	0.3	3	2.2	0.005
Lemnetum minoris	2	7.7	0.01	13	9.4	0.04
Lemnetum trisulcae	1	3.8	0.02	7	5.0	0.1
Class: **Charetea**						
Charetum tomentosae	22	84.6	15.2	32	23.0	2.5
Nitellopsidetum obtusae	19	73.1	8.2	28	20.1	2.1
Charetum rudis	17	65.4	9.0	15	10.8	1.3
Charetum fragilis	17	65.4	8.3	25	18.0	0.8
Charetum contrariae	11	42.3	0.8	18	12.9	0.6
Nitelletum flexilis	6	23.1	0.7	6	4.3	0.2
Charetum filiformis	6	23.1	0.6	1	0.7	0.001
Charetum delicatulae	5	19.2	2.2	4	2.9	0.2
Charetum asperae	5	19.2	0.7	7	5.0	0.2
Charetum intermediae	3	11.5	0.6	3	2.2	0.5
Charetum hispidae	2	7.7	0.1	-	-	-
Charetum polyacanthae	1	3.8	0.02	-	-	-
Charetum vulgaris	-	-	-	1	0.7	0.02
Lychnothamnetum barbatii	-	-	-	1	0.7	0.1
Class: **Potametea**, Alliance: **Potamion**						
Potametum lucentis	21	80.8	4.5	54	38.8	2.1
Ceratophylletum demersi	18	69.2	4.3	73	52.5	10.1
Potametum perfoliati	18	69.2	0.4	76	54.7	1.7
Myriophylletum spicati	17	65.4	5.1	63	45.3	4.1

Plant communities	Reference lakes (n=26)			Non-reference lakes (n=139)		
	No of occurrence	Frequency (% of lakes)	Mean cover (%)	No of occurrence	Frequency (% of lakes)	Mean cover (%)
Ranuncutetum circinati	16	61.5	1.6	59	42.4	1.9
Elodeetum canadensis	13	50.0	0.4	44	31.7	2.7
Potamo-Najadetum marinae	8	30.8	1.2	10	7.2	0.1
Potametum pectinati	8	30.8	0.1	39	28.1	1.3
Myriophylletum verticillati	6	23.1	1.0	5	3.6	0.03
Potametum friesi	4	15.4	0.6	29	20.9	2.4
Potametum filiformis	3	11.5	0.03	5	3.6	0.03
Potametum obtusifoli	3	11.5	0.03	1	0.7	0.02
Potametum compressi	2	7.7	0.04	16	11.5	0.3
Ass. with *Potamogeton crispus*	2	7.7	0.01	10	7.2	0.04
Ass. with *Potamogeton pusillus*	-	-	-	6	4.3	0.2
Potametum filiformis f. with *Potamogeton praelongus*	-	-	-	5	3.6	0.02
Ceratophylletum submersii	-	-	-	4	2.9	0.7
Zannichellietum palustris	-	-	-	2	1.4	0.1
Potametum filiformis f. with *Potamogeton alpinus*	-	-	-	1	0.7	0.02
Potametum graminei	-	-	-	1	0.7	0.02
Potametum nitens f. with *Potamogeton rutilus*	-	-	-	1	0.7	0.004
Ass. with *Potamogeton trichoides*	-	-	-	1	0.7	0.001
Class: **Potametea**, Alliance: **Nympheion**						
Nupharo-Nymphaeetum f. with *Nuphar lutea*	21	80.8	2.8	82	59.0	5.3
Stratiotetum aloidis	18	69.2	0.8	48	34.5	1.2
Potametum natantis	13	50.0	0.4	36	25.9	0.6
Nupharo-Nymphaeetum f. with *Nymphaea alba*	10	38.5	0.4	25	18.0	0.6
Polygonetum natantis	5	19.2	0.1	33	23.7	0.1
Hydrocharitetum morsus-ranae	1	3.8	0.004	15	10.8	0.2
Class: **Phragmitetea**, Alliance: **Phragmition**						
Phragmitetum australis	25	96.2	26.2	120	86.3	28.1
Scirpetum lacustris	21	80.8	1.6	85	61.2	1.4
Typhetum angustifoliae	19	73.1	2.3	96	69.1	4.2
Typhetum latifoliae	19	73.1	0.8	84	60.4	1.6
Equiseteum fluviatilis	17	65.4	0.2	44	31.7	0.3
Sparganietum erecti	16	61.5	0.2	71	51.1	0.5
Acoretum calami	9	34.6	0.04	59	42.4	1.6
Glycerietum maximae	5	19.2	0.03	76	54.7	2.5
Cladietm marisci	1	3.8	0.02	5	3.6	0.1
Scirpetum tabernaemontani	1	3.8	0.004	6	4.3	0.01
Scirpetum maritimi	-	-	-	3	2.2	0.005
Class: **Phragmitetea**, Alliance: **Oenanthion**						
Eleocharitetum palustris	14	53.8	0.2	65	46.8	0.3
Sagittario-Sparganietum f. with *Sagittaria sagittifolia*	3	11.5	0.01	13	9.4	0.02
Sagittario-Sparganietum emersi f. *Sparganium emersum*	3	11.5	0.01	13	9.4	0.2
Butometum umbellati	-	-	-	8	5.8	0.1
Rumex hydrolapathum	-	-	-	2	1.4	0.1
Alisma lanceolatum	-	-	-	2	1.4	0.001
Oenantho-Rorippetum	-	-	-	2	1.4	0.001
Class: **Phragmitetea**, Alliance: **Magnocaricion**						
Caricetum rostratae	23	88.5	0.4	58	41.7	0.9
Caricetum acutiformis	17	65.4	0.4	57	41.0	1.0
Thelypteridi-Phragmitetum	16	61.5	0.9	43	30.9	1.1

Plant communities	Reference lakes (n=26)			Non-reference lakes (n=139)		
	No of occurrence	Frequency (% of lakes)	Mean cover (%)	No of occurrence	Frequency (% of lakes)	Mean cover (%)
Caricetum ripariae	13	50.0	0.2	44	31.7	0.5
Phalaridetum arundinaceae	6	23.1	0.1	29	20.9	0.1
Caricetum paniculatae	5	19.2	0.1	17	12.2	0.04
Caricetum gracilis	2	7.7	0.1	14	10.1	0.1
Caricetum appropinquatae	2	7.7	0.01	4	2.9	0.01
Caricetum lasiocarpae	2	7.7	0.01	1	0.7	0.001
Caricetum vesicariae	2	7.7	0.01	6	4.3	0.03
Cicuto-Caricetum pseudocyperi f. with *Carex pseudocyperus*	2	7.7	0.01	7	5.0	0.01
Cicuto-Caricetum pseudocyperi f. with *Cicuta virosa*	2	7.7	0.01	8	5.8	0.01
Iridetum pseudacori	1	3.8	0.02	15	10.8	0.01
Sparganio-Glycerietum fluitantis	1	3.8	0.02	2	1.4	0.004
Caricetum elatae	-	-	-	9	6.5	0.2
Ass. with *Lysimachia thyrsiflora*	-	-	-	10	7.2	0.04
**Other**						
Ass. with *Fontinalietum antipyreticae*	4	15.4	0.1	20	14.4	0.3
Ass. with *Platyhypnidium riparioides*	2	7.7	1.2	-	-	-
Ass. with *Pseudocalliergon lycopodioides*	1	3.8	0.004	-	-	-

References

Brzeg, A., & Wojterska, M. (2001). Plant communities in Wielkopolska: the state of knowledge and threats. In M. Wojterska (Ed.), Flora and vegetation of Wielkopolska and South-Pomerania Lakeland. Guide book of 52. Reunion of Polish Botanical Society, Poznan, 39–110 (in Polish).

Matuszkiewicz, W. (2002). A guide book to identification plant communities in Poland. PWN, Warszawa (in Polish).
